# Unraveling Emergency Room Patterns: Advancing Acute Care in a Newly Established Tertiary Hospital

**DOI:** 10.7759/cureus.85993

**Published:** 2025-06-14

**Authors:** Rashmi Ranjan Guru, Namrata Mathur, Soujanya Kumar, Meenakshi Sharma, Swayamprava Dalai, Subhodip Mitra

**Affiliations:** 1 Hospital Administration, All India Institute of Medical Sciences, Jodhpur, Jodhpur, IND; 2 Emergency Medicine, All India Institute of Medical Sciences, Jodhpur, Jodhpur, IND; 3 Hospital Administration, Government Medical College, Anantapur, Anantapur, IND; 4 Nursing, Postgraduate Institute of Medical Education and Research (PGIMER), Chandigarh, IND; 5 Biotechnology, Chandigarh University, Mohali, IND; 6 Hospital Administration, All India Institute of Medical Sciences, Kalyani, Kalyani, IND

**Keywords:** emergency cases, emergency patient care, management of emergency resources, patient profile, quality improvement

## Abstract

Emergency conditions are unavoidable, critical health conditions requiring immediate intervention and treatment. Emergency treatment in our country is quite unregulated and below the benchmark, which may be due to untrained medical staff, bad transportation facilities, and inadequate preparedness. Many emergency cases in our country could not even reach the hospital. In rural areas, primary and community health centers are generally not sufficient to provide adequate emergency services, considering the low ratio of staff, equipment, and lifesaving drugs. Road traffic accidents, acute myocardial infarction (AMI), and cerebrovascular accidents (CVAs) are nowadays taking many lives due to a lack of golden hour treatment. Cardiac arrest cases cannot survive due to a lack of knowledge about defibrillation. AMI cases are neglected in primary centers due to a lack of drugs and electrocardiography (ECG) machines, and patients are unable to survive due to delayed treatment. Upgrading emergency services is essential based on the type of cases visiting the emergency room.

The study highlighted the primary nature of cases presented to the emergency department (ED), emphasizing the importance of a well-structured, responsive emergency care system. By identifying common trends, peak hours, and the types of emergencies coming to the hospital, this study provides valuable insights into resource allocation, staffing needs, and the development of protocols. The study’s findings ensure the efficiency and effectiveness of emergency services, ultimately improving patient outcomes in the golden hour of treatment and reducing waiting times in the emergency department. Continuous surveillance and periodic evaluation of emergency case patterns will help make a resilient emergency care system.

## Introduction

Emergency conditions are unavoidable, critical health conditions requiring immediate intervention and treatment. Recent statistics of mortality in India in emergency rooms are nearly 43,939.49 per one lakh of inpatient deaths [1]. Emergency treatment in a country like India is quite unregulated and below the benchmark due to untrained medical staff, bad transportation facilities, and inadequate preparedness. In rural areas, primary and community health centers are generally not sufficient to provide adequate emergency services, considering the low ratio of staff, equipment, and lifesaving drugs to the large population. Road traffic accidents, acute myocardial infarction (AMI), and cerebrovascular accidents (CVAs) are nowadays taking many lives due to a lack of golden hour treatment [2]. In the early years of the 20th century, fewer patients used to attend the emergency department (ED). Nowadays, emergency rooms are becoming crowded with medical and trauma cases, with medical emergencies contributing a significant part. In low-income countries with limited resources to provide quality patient care to a large number of people, the requirement of an emergency department is crucial. The death rate per 1,000 emergency department admissions in 2021 was 2.786, while in the first nine months of 2022, it was 2.619 [3].

Overcrowding in the emergency department (ED) is becoming a concern, as it contributes to poor quality, increased morbidity and mortality, and poor staff morale. Access blocks, lack of beds, and inadequate disposal planning are common issues in the ED [4]. To manage the increased workload from the different clinical profiles of patients and for their management, resource optimization is one of the most important parameters in the emergency department [5,6]. Point-of-care testing has improved the diagnosis time. For example, some patients can be discharged from the emergency department with low-risk chest pain cases, leading to decreased length of stay in the emergency department [7].

The objective of the study was focused on the improvement of the quality of services provided in the emergency department by efficient resource utilization.

## Materials and methods

Study setup

The study was a cross-sectional, retrospective study conducted for three months in the year 2024, from February to April, in the emergency room of a newly functional teaching hospital in India named Symbiosis University Hospital and Research Centre, Pune.

Study population

We included 2,651 patients who attended the emergency department during the study period from February to April 2024. All adult patients who attended the emergency department during the study period were included in the study. Patients with chronic disease were excluded from the study population.

Data collection and analysis

Data regarding patient complaints, treatment, and proceedings were obtained from the emergency records. The authors collected the data from the hospital information management system (HIMS). Data were analyzed using Statistical Package for the Social Sciences version 16.0 (SPSS Inc., Chicago, Ill) to find the different categories of patients attending the emergency room, and descriptive statistics were used to find out the most common cases.

Data protection and ethics

The study was a quality improvement study, and the authors collected observational data from the hospital information management system (HIMS). Access to data using appropriate computer equipment (approved equipment or via agreed remote desktop access) by the institute’s HIMS was maintained. Statistical Package for the Social Sciences version 16.0 was used to calculate percentages, and logistic regression analysis was done while keeping free from possible data breaches. All data were saved in a secure location on a secure drive within the organization’s HIMS. All data curation, handling, and dissemination align with local data policies and comply with the Data Protection Act 2018.

## Results

Data were collected from the emergency register and the hospital information management system; 2,651 patients had come to the emergency room in three months, from February to April 2024. The gender and age distribution of emergency cases is shown in Table 1.

**Table 1:** Gender and age distribution of emergency cases (N=2,651)

Variables	Number (%)
Gender	
Male	1,466 (55.3%)
Female	1,185 (44.7%)
Age groups	
Infant	103 (3.9%)
Child	472 (17.8%)
Adult	1,779 (67.1%)
Geriatric	297 (11.2%)

A total of 2,651 cases were divided into medicine and allied specialties and surgery and allied specialties, as shown in Table 2. Fever was the common complaint, with the highest number of patients (795 (30%)), followed by acute gastroenteritis (477 (8%)).

**Table 2:** Percentage of different emergency cases coming to the emergency room at the tertiary care hospital (N=2,651) CVA: cerebrovascular accident, AMI: acute myocardial infarction, COPD: chronic obstructive pulmonary disease

Type of emergency case	Number (%)
CVA cases	53 (2%)
Suicidal cases (hanging and poisoning)	53 (2%)
Obstetric emergency cases	79 (3%)
Dehydration	132 (5%)
Miscellaneous	159 (6%)
AMI/chest pain	212 (8%)
Abdominal pain	212 (8%)
Orthopedic fractures/injury/back pain	212 (8%)
Pneumonia/COPD/asthma	265 (10%)
Acute gastroenteritis/gastritis	477 (18%)
Fever	795 (30%)

Out of 2,651 cases, emergency procedures were performed on 1,561 (58.9%) patients. The most common procedures were intravenous (IV) catheter placement (1,264 (47.7%)), followed by electrocardiography (ECG) (755 (28.5%)). The most common tests requested were complete blood count (CBC) (1,426 (53.8%)) to find out the severity of infection, followed by X-rays (683 (25.8%)) to find out fractures in bones in orthopedic cases and pneumonia in cases that presented with shortness of breath. The most commonly requested consultations were from the internal medicine, cardiology, and surgery departments, and the numbers and percentages were described in Table 3.

**Table 3:** Utilization of services in the emergency room (N=2,651) IV: intravenous, ECG: electrocardiogram, ABG: arterial blood gas

Variables	Number (%)
Most common consultations in the emergency room	
Internal medicine	723 (27.3%)
Cardiology	182 (6.9%)
Surgery	151 (5.7%)
Pediatrics	98 (3.7%)
Most common laboratory tests	
Complete blood count	1,426 (53.8%)
Creatinine	1,047 (39.5%)
Most common imaging studies	
X-ray	683 (25.8%)
Most common procedures	
IV placement	1,264 (47.7%)
ECG	755 (28.5%)
Wound care	371 (1.4%)
Suturing	265 (10.0%)
ABG	212 (8%)

## Discussion

In this study, after data analysis, it was found that among medicine and allied cases, fever, diarrhea, gastritis, shortness of breath, and acute chest pain were very common. Among the surgical and allied cases, orthopedic emergencies and abdominal pain most often came to the emergency room. Obstetrics and neurological cases were common occurrences in this study, which had a similar finding in quality of care documented by the World Health Organization (WHO) [8]. With the availability of adequate resources, particularly equipment and laboratory services, the golden hour of treatment became more fruitful. Resource utilization as per the need for the cases coming to the emergency provided an optimum environment for the safety of the patient as well as the doctors and staff. Barrier safety measures are to be provided to the staff during emergency work, as the past history of chronic disease (hepatitis B virus (HBV), hepatitis C virus (HCV), and human immunodeficiency virus (HIV)) status of the patient was not known, as mentioned by another author [9].

Training of staff for lifesaving procedures in the emergency room, such as basic life support (BLS) and advanced cardiac life support (ACLS), along with mock drills, is to be done on a monthly basis to update the knowledge of the staff and to reduce the decision time in lifesaving procedures, similar to other study sources [10-13]. An emergency medical team (EMT) with ambulance service is important for the emergency department to rescue patients in emergency situations. The EMT ideally consists of trained pharmacists, a trained nurse, trained hospital attendants, and a trained ambulance driver. The ambulance should be fully equipped with emergency drugs, an oxygen cylinder, a ventilator, a bilevel positive airway pressure (BiPAP), a nebulizer, and IV fluids, along with a telecommunication system in emergency situations, as mentioned in another study [14]. In this study, the EMT has proven to be an important part of the functioning of the system by enlisting the emergency lifesaving drugs, and their easy handling is an important part of inventory management in the emergency room. A crash cart is to be kept ready in an emergency room to bring all the emergency drugs at once, along with the ECG machine to take care of the platinum minutes for the patient’s survival, as mentioned in other studies [7].

Acute myocardial infarction (AMI) is a life-threatening situation. The stress factor in educational institutes and food habits aggravates the condition. The ECG machine facility is crucial for the diagnosis of AMI. Emergency drugs such as oral aspirin (325 mg), oral clopidogrel (150-300 mg), oral atorvastatin (80 mg), oral metoprolol (50 mg), and injections of low-molecular-weight heparin or injections of heparin must be kept in the emergency department; this is not mentioned in any case study but is followed in every hospital with some deficiencies in the practice of the pharmacy inventory process. Road traffic injuries (RTIs) and traumatic sports injuries coming to the emergency department are to be treated as soon as possible (golden hour treatment); this is to be kept as a standard operating procedure (SOP), and skill development of the staff is to be developed to follow the SOP. An SOP for medicolegal cases (MLCs) is to be followed, and an injury report is to be written for medicolegal purposes. Blood transfusion protocols in cases of hemorrhagic shock, hemostasis achievement, application of pelvic binder, and fracture stabilization are to be followed in the emergency room, as mentioned in a study conducted by the All India Institute of Medical Sciences, New Delhi, India [14]. In this study, we found that dehydration and heatstroke cases were the most common during the summer season. Therefore, intravenous fluids such as dextrose in normal saline (DNS), normal saline (NS), Ringer’s lactate (RL), 25% dextrose in water (25%D), 10% dextrose in water (10%D), and 5% dextrose in water (5%D) are to be kept in emergencies (not mentioned in any studies). Obstetric emergencies, such as postpartum hemorrhage, antepartum hemorrhage, placenta previa, eclampsia, and abruptio placentae, are serious concerns that need immediate treatment, and appropriate technology and infrastructure are important concerns and are followed in most parts of the world to decrease neonatal mortality and maternal mortality rates. Neurological cases such as cerebrovascular accidents, either ischemic or hemorrhagic, come to the emergency room very often, and it is extremely important to provide treatment in the golden hour for such cases to improve the long-term disability, as mentioned in literature from the case studies in emergency medicine [15]. Finally, manpower management is the main concern, including doctors, paramedical staff, hospital attendants, and housekeeping staff, who play important roles in the smooth operation of the emergency room (not mentioned in any other study).

The National Accreditation Board for Hospitals and Healthcare Providers (NABH) has provided emergency models for the setup of emergency departments in hospitals, along with SPOs for the smooth operation of the emergency department [16].

Limitations

The study is limited to a single hospital and has a specific time frame, limiting the findings of the healthcare setup. Selection bias exists, as usually, serious patients come to the emergency room. Emergency cases often involve incomplete patient details and missing medical records, which may affect the accuracy of data. For study in the emergency room, there may be a requirement for additional ethical approvals and obtaining informed consent from the patients, which were significant limitations to this study. Resource allocation in an emergency may influence the results of the study.

Recommendations

Periodic assessments of potential risks, including violence, are to be conducted, and regular reports must be submitted to the authorities. The documented responsibilities of the staff for handling disaster situations must be displayed on the noticeboard. Standard operating procedures (SOPs) for emergency codes to manage emergency situations are to be maintained in the emergency room. Female staff are posted in pairs at night shifts or in isolated wards. Safety doors and windows with grills and wire mesh must be secured. Entry of vendors and hawkers into the premises must be restricted. A control room is to be established for the coordination of the activities of the emergency department.

## Conclusions

The study highlighted the primary nature of cases that presented to the emergency department, highlighting the importance of a well-structured emergency care system. By identifying common trends, peak hours, and the types of emergencies coming to the hospital, this study provides valuable insights into resource allocation, staffing needs, and the development of protocols. The study identified fever as the most common occurrence in the study place, followed by shortness of breath and chest pain-related emergencies. The study provided an example to illustrate the practical significance of determining peak hours, and this can aid in shift scheduling optimization and guarantee the proper distribution of specialized personnel and equipment during times of high demand.

As a further recommendation, a biannual review cycle that utilizes a standardized data capture tool and dashboard analytics must also be put in place. This approach will allow for timely adjustments in resource planning and staffing. Continuous surveillance and periodic evaluation of emergency case patterns will help make a resilient emergency care system.
